# A Critical Analysis of COA Research

**Published:** 1997

**Authors:** Mary Beth de Ribeaux

**Affiliations:** Mary Beth de Ribeaux, a science editor of Alcohol Health & Research World, coordinated the panel discussion

**Keywords:** children of alcoholics, research, familial alcoholism, historical overview, AOD use susceptibility, hereditary factors, environmental factors, behavioral and mental disorder, high risk group, risk assessment, risk factors, etiology, controlled study, longitudinal study, comparative study, biological markers

## Abstract

Five experts respected for their significant contributions to the scientific literature on children of alcoholics (COA’s) offer their perspectives in a panel discussion format. The panel members reflect on the historical roots of COA research and comment on its current status and future direction. Enriched by the panelists’ variety of backgrounds, research interests, and approaches, the discussion emphasizes the need to consider multiple variables that influence the risk for alcoholism among COA’s.

Over the past 20 years or so, much has been written about children of alcoholics (COA’s) in the scientific, clinical, and popular literature. Within this diverse collection of published material, authors discussing the nature and treatment of COA’s sometimes have put forth controversial generalizations and assertions that are upheld by various degrees of research-supported empirical evidence. For example, COA’s frequently are labeled as “codependents,” but a standard set of diagnostic criteria for codependency^1^ has not been established in the professional community, and research to date has not determined whether COA’s are codependent at a higher rate than any other special population or the population in general. Similarly, the personality attributes often ascribed to COA’s (e.g., self-blame, anger, and excessive need to control) do not characterize all COA’s and may not be unique to this group (i.e., such characteristics may describe anyone growing up in a stressful environment).

To help sort fact from speculation and put COA issues into better focus, *Alcohol Health & Research World* (*AH&RW*) invited a select panel of experts known for their groundbreaking scientific work related to COA’s to comment on the current status and direction of research in this area. We are pleased to present the perspectives of the following panel members, whose viewpoints have been shaped by a variety of research interests and backgrounds:

- *Laurie Chassin, Ph.D.*—professor of psychology in the psychology department at Arizona State University, Tempe, Arizona.
- *Theodore Jacob, Ph.D.*—career research scientist at the Palo Alto Veterans Administration Health Care System, Palo Alto, California.
- *Jeannette L. Johnson, Ph.D.*—associate professor of psychiatry and director of substance abuse research and program evaluation in the Department of Psychiatry, Division of Alcohol and Drug Abuse, University of Maryland at Baltimore, Maryland.
- *Marc A. Schuckit, M.D.*—professor of psychiatry at the University of California–San Diego, School of Medicine, and director of the Alcohol Research Center, Veterans Affairs Medical Center, San Diego, California.
- *Kenneth J. Sher, Ph.D.*—Frederick A. Middlebush Professor of Psychology in the Department of Psychology at the University of Missouri, Columbia, Missouri.

***AH&RW:**** Where has COA research come from? What is it rooted in and what was it a response to?*

**Sher:** This is a good question, and I’m not sure there is a single answer, because COA’s have been studied for a number of different reasons over the years, with little obvious linkage among different threads of inquiry. It is clear that problems associated with alcoholic parents have been recognized for hundreds of years. From a quantitative perspective, however, the earliest work on this topic of which I am aware dates to the beginning of the current century, when a spirited debate occurred between Karl Pearson, a leading psychologist and statistician, and J. M. Keynes, the noted Cambridge economist, concerning the extent to which the offspring of alcoholics were characterized by intellectual deficits. It is instructive that Keynes’ methodological critique of Pearson’s work echoes many criticisms of contemporary investigations, such as the effects of sampling on obtained findings.

**Johnson:** I agree with Dr. Sher that COA research has a long history preceding the studies that have been published in the last several decades. Some of the earliest COA research was published at the turn of the century in the *Journal of Inebriety*, and Margaret Cork’s 1969 book, *The Forgotten Children*, can be heralded as one of the earliest studies of behavior in COA’s.

Aside from this early work indicating interest in the plight of COA’s, however, I think we can trace three separate influences on contemporary studies. First, a substantial amount of research has been conducted in an attempt to understand the intergenerational transmission of alcoholism. Many of those early studies used the Scandinavian population registers to gather data about the relationship of parental alcoholism to biological children who were either living with their biological parent or had been adopted. Second, at about the same time that researchers were conducting studies to determine the incidence of alcoholism across generations, Norman Garmezy, together with his students and colleagues, was asking similar questions about the transmission of psychopathology, especially schizophrenia. Garmezy’s work is partially responsible for shaping the early research on children at risk for alcoholism. His research entailed a difficult method of prospectively studying children born to and living with a parent with a chronic mental disorder (i.e., observing the children over a long period of time). Many researchers studying COA’s adapted this same research method and asked questions similar to those that had been previously asked in the mental health field—namely, who is at risk and what determines the predisposition to express this risk? Third, the early COA public advocacy movement had a tremendous influence on research with COA’s in the United States because of the movement’s enormous impact on the public, which in turn influenced the way Congress allocated research dollars.

**Schuckit:** The work by Garmezy mentioned by Dr. Johnson is part of a long tradition in psychiatric and medical research of trying to identify people who are vulnerable to a particular disorder and to follow them over time. For example, longitudinal studies have tried to identify people at high and low risk for heart disease as well as schizophrenia. Once such populations are identified, researchers attempt to determine the characteristics of people in these groups that lead to risk (or resilience). As Dr. Johnson points out, studies of COA’s grew out of this general tradition. Researchers seeking to identify people at high risk for alcoholism focused on COA’s, because studies showed that close relatives of alcoholics have a fourfold higher risk for alcoholism. Although both environmental and genetic factors appear to play a role in alcoholism, we know that alcoholism is at least partly genetic, because of the findings from both adoption and twin studies. Even if a person with a family history of alcoholism is adopted and raised by nonalcoholics, the fourfold higher risk still holds.

**Jacob:** Although several lines of clinical and scientific inquiry have contributed to current interest in the study of COA’s (see Sher 1991 and Seilhamer and Jacob 1990 for brief historical overviews), I agree that the pioneering studies of schizophrenia associated with the work of Garmezy (as well as Rodnick, Mednick, Wynn, Goldstein, and so forth) were a particularly important stimulus. Studies such as these, which emerged during the early 1950’s in various subareas of psychopathology research, used a “high-risk” design. Briefly, the high-risk research strategy focuses on populations at increased risk for developing a disorder (such as schizophrenia or alcoholism) in order to maximize theoretical precision and research resources by ensuring that a reasonably large number of subjects within a designated sample eventually develop the disorder. The critical implication of this strategy is that it encourages researchers to study the development of the disorder over time and to systematically compare people who exhibit the particular psychopathology under investigation with people who do not. Researchers in the alcohol field have long recognized that alcoholism is a strongly familial disorder, as Dr. Schuckit noted, and that COA’s are clearly at much greater risk for alcoholism than are offspring of nonalcoholic parents. Therefore, the study of COA’s as high-risk subjects became a particularly important strategy for studying the etiology and development of alcoholism.

**Chassin:** I think there are multiple roots of COA research, which is appropriate for such a multifaceted research area. First, and most basic, COA studies are fueled by research interest in understanding the etiology of alcoholism. As Dr. Jacob commented, for researchers interested in alcoholism risk markers and alcoholism etiology, COA’s are the obvious group to study, because their risk is well established and they can be identified fairly easily. In the tradition of high-risk research more broadly, the advantage of COA studies is that characteristics of COA’s can be identified that predate the onset of alcoholism. In other words, studying COA’s can help distinguish between the antecedents and consequences of alcoholism. The question of intellectual deficits mentioned by Dr. Sher is one example. Studying COA’s can help determine whether any intellectual deficits precede alcoholism (and raise the risk for alcoholism) as opposed to intellectual deficits that occur as a result of heavy alcohol consumption.

A second part of the foundation for COA research springs from interest in the effects of alcohol exposure on fetal development. The literature on fetal exposure brings a unique perspective to COA research, particularly because it focuses on the effects of maternal alcoholism, which is understudied in other COA work. Lastly, a third important underpinning of COA research is a broad clinical concern for the mental health outcomes of COA’s. This tradition began with an early recognition that COA’s are at risk for a wide range of mental health problems in addition to alcoholism. The tradition also has maintained an active interest in intervention programs to improve COA outcomes.

***AH&RW:**** How did you, specifically, become interested in this research area?*

**Chassin:** I’m not sure that any of the three traditions I just described exactly capture my own interest in COA research. I came to this area with a background as a child clinical psychologist interested in adolescent problem behavior, particularly adolescent substance use, and I wanted to apply life span developmental perspectives to longitudinal studies of substance use initiation. My prior research experience was with a large community study of tobacco use, which is still ongoing. At the time (the mid-1980’s), I started to think that large general population studies of adolescent substance use might be missing important data, because the subgroup at highest risk was often underrepresented in the samples, particularly in large school-based surveys. Around the same time, I also had the opportunity to do some collaborative work with Ken Sher, which allowed me to learn more about COA research. I thought that COA’s were an ideal high-risk group to study, because they offered me a chance to connect what I knew about the etiology of substance use in the general adolescent population with the development of more serious substance use problems. For me, conducting longitudinal research with COA’s allows for a developmental psychopathology perspective. That is, research on COA’s as a high-risk group forms a bridge between the study of substance use disorders and the study of adolescent development more broadly.

**Schuckit:** I became involved in COA research in the early 1970’s. We knew by 1972 that alcoholism appeared to be genetically influenced (Schuckit et al. 1972), and I wanted to know more about what traits or characteristics might be inherited to increase the risk. Prospective studies of COA’s, which follow large groups over a long period, seemed to be the most logical way to go about this.

**Sher:** My personal interest in COA research began in graduate school in the late 1970’s when I became aware of the high-risk study method previously mentioned, which was being promoted by Mednick and McNeill for the study of schizophrenia. My opinion at the time, which remains my opinion 20 years later, is that the high-risk design (in which the offspring of alcoholics are contrasted with the offspring of nonalcoholics) is potentially more useful for the study of alcoholism than it is for the study of schizophrenia. First, unlike schizophrenia (in which only 10 percent of schizophrenics have an affected parent), a substantial proportion of alcoholics (perhaps 30 to 50 percent) have alcoholic parents. Thus, the findings from high-risk studies are likely to be more generalizable to alcoholism.

Second, and probably more important, alcoholism is one of the few behavior disorders in which a necessary condition for the development of the disorder is known—namely, the use of alcohol. I reasoned that COA’s are an identified group with presumed vulnerability and that we could test how members of this group differ from other people in response to a known etiological agent (i.e., alcohol). Marc Schuckit and Bob Pihl clearly have done more than anyone else in exploring this idea. While in graduate school, I was very much influenced by a theoretical review written by Ralph Tarter in 1978 that, to my mind, represents a seminal piece on the vulnerability to alcoholism. Tarter’s theoretical work was anticipated by a number of creative theorists and experimentalists, including William McDougall in the 1920’s, Benjamin Kissin and the late Hans Eysenck and his colleagues in the 1950’s, and Petrie in the 1960’s. However, the emerging findings on the heritability of alcoholism by Goodwin and colleagues in Denmark gave much-needed momentum to the study of alcoholism vulnerability. In the past 20 years, the field has matured markedly with the work of a number of distinguished scientists, including Bob Cloninger, Henri Begleiter, Laurie Chassin, Andrew Heath, Bob Pihl, and Marc Schuckit, to name just a few.

**Jacob:** My own interests in COA research emerged during the late 1970’s as well, when I began a large-scale, longitudinal study of families containing an alcoholic parent. Initially the study focused on providing a systematically constructed database on the nature of relationships (including marital as well as parent-child) in families in which alcoholism occurs and contrasting these patterns with patterns in families containing other types of psychiatric disorders. During the past decade, the study’s major interests have shifted to focus on the development of the offspring in families with alcoholic parents and in clarifying the nature of family- and individual-level influences relevant to the development of alcoholism among the children, who are now young adults.

**Johnson:** I became involved in COA research shortly after I received my Ph.D. I had been working for Monte Buchsbaum doing brain imaging research with schizophrenics, and after I completed my dissertation on evoked potentials in his laboratory, I looked for another position. As part of its Intramural Research Program, NIAAA [the National Institute on Alcohol Abuse and Alcoholism] had just created the Laboratory of Clinical Studies under the leadership of Markku Linnoila, with whom I had collaborated in Monte’s lab. Markku was interested in brain imaging, needed some assistance, and offered me a job. I agreed to take the brain imaging position, but asked whether I could continue my own research interests with children. Markku agreed, and thus I started COA research in the new NIAAA program purely by chance.

***AH&RW:**** What is the state of COA research today? How has it evolved?*

**Schuckit:** COA research is now incredibly more sophisticated than it was in its early days, and this sophistication has increased dramatically in the last 5 to 10 years. Several reasons explain this progress. For one, more people recognize the importance of biological factors in the increased risk for alcoholism. In addition, some of the characteristics of at-risk children have become known, and that knowledge opens up a host of possibilities using other research approaches, such as electrophysiological testing to identify who is at risk (as done by Henri Begleiter, Bernice Porjesz, Cindy Ehlers, and Shirley Hill).

Overall, COA research today might be divided into two categories. First, cross-sectional studies compare COA’s with control subjects (i.e., non-COA’s), who are as similar as possible to the COA’s except that they do not have a family history of alcoholism. The high- and low-risk groups are compared at one point in time in an attempt to identify characteristics of COA’s that might be related to their risk for developing alcoholism. Cross-sectional studies have generated some strong research leads by showing that COA’s and non-COA’s differ on a variety of characteristics. For instance, in a study I did with Tom Smith (Schuckit and Smith 1996), COA’s and non-COA’s differed in the intensity of their response to alcohol. Generally, COA’s seem to be able to “hold their liquor” well compared with non-COA’s in controlled studies. Other laboratories have been interested in identifying differences in additional domains. Some studies have shown variations between COA’s and non-COA’s in styles of reasoning, for example. Although these findings are interesting, they are difficult to interpret. Electrophysiological differences between COA’s and non-COA’s are another example. Some of these differences relate to factors that impact characteristics such as impulsivity, and some very interesting leads have emerged in this area. Personality test measures also differ in COA’s and non-COA’s, primarily those that gauge impulsivity and boredom susceptibility (i.e., how easily a person becomes bored). In addition, some very technical chemical findings have come forth, such as variations in the activity levels of the enzyme monoamine oxidase, which plays a role in the breakdown of important neurotransmitters thought to influence mood and behavior. All in all, a host of cross-sectional studies in the past 5 to 10 years have led to a more sophisticated understanding about how COA’s and non-COA’s differ.

A second category of COA research involves longitudinal studies. These investigations follow up on the findings from cross-sectional results to see whether the risk-related characteristics identified in those studies actually predict alcoholism. To my knowledge, only three major longitudinal COA studies currently exist, of which ours (Schuckit and Smith 1996) is the largest. In our work, 450 out of 453 men (99.3 percent) were followed successfully for 10 years, with data revealing that a low level of response to alcohol at about age 20 was a very good predictor of alcoholism by about age 30.

Response to alcohol does not operate alone, however, and during our 15-year followup, we are interested in finding other characteristics that might add to or detract from the ability of the low response to alcohol to lead to alcoholism. These other factors might include a person’s expectancies about the effects of alcohol, levels of life stress, stress coping style, personality characteristics, and so forth. It is no longer acceptable to look at one characteristic alone, and we are attempting to simultaneously measure as many aspects of a person’s life as possible.

An additional factor that has increased the sophistication of COA research is the availability of new (and sometimes expensive) techniques to perform these studies, using advances in brain imaging, neurochemistry, and statistical methods. The overall result has been a nearly exponential increase in study sophistication, making COA research an exciting field to be in.

**Jacob:** Examination of the current scientific literature on COA’s indicates increasing interest in several features: (1) conducting theoretically driven work on the nature and severity of impairments exhibited by COA’s; (2) implementing longitudinal designs to describe the developmental course of COA’s more systematically; (3) incorporating a range of potentially influential variables (including individual, contextual/environmental, and family relationship variables) that may predict COA development and outcomes; and (4) employing greater use of powerful, statistical-analytic strategies for testing emergent models.

A particularly notable milestone in this literature involves the increasing emphasis on describing mediators and moderators of risk and testing models concerned with how the risk-outcome relationship is explained (i.e., mediated) or qualified (i.e., moderated). To a significant extent, this work has been spearheaded by the theoretical and empirical studies of Sher and colleagues (Sher 1991; Sher et al. 1991; Sher et al. 1996) as well as the important contributions of several other longitudinally based research programs (e.g., Chassin et al. 1993; Newcomb and Felix-Ortiz 1992). Most of these efforts have focused on three general mediator mechanisms (i.e., deviant socialization, affect regulation, and pharmacologic vulnerability^2^), each based on a model developed from existing theoretical and empirical studies.

**Chassin:** One way of describing “modern” COA research is by noting some common themes that have emerged in several recent important publications. Sher (1991) and Windle and Searles (1990) assessed the state of COA literature and pointed out directions for future research, and Zucker (1994) proposed a developmental perspective on COA risk. The research agenda set out in these publications illustrates many of the features (or, at least, aspirations) of current COA studies. Contemporary studies also are characterized by numerous methodological improvements. For example, researchers are paying greater attention to sampling strategies (e.g., by including samples drawn from the community in addition to samples from treatment facilities or school-based samples), the heterogeneity of parent alcoholism (e.g., the incidence of co-occurring disorders in parents and multigenerational alcoholism), and age and sex variations in COA outcomes. As another noteworthy improvement, researchers have expanded the guiding conceptual frameworks of their studies to consider biopsychosocial models. Rather than positing a competition between biological and environmental causes, investigators are now accepting the need to examine the interrelation and interaction among multiple risk factors and levels of analysis. We are recognizing that numerous pathways underlie risk and resilience. Researchers also are conducting more longitudinal studies that use a developmental framework to assess outcome models with multiple variables. In addition, researchers are taking advantage of innovations in quantitative methods to test these complex, multivariate, longitudinal models more appropriately. Finally, recent studies do not proceed from the assumption that negative outcomes for COA’s are universal and inevitable. Rather, studies are beginning to identify the mediating mechanisms that are responsible for negative outcomes as well as the protective factors that buffer risk for COA’s.

**Johnson:** The literature is awash with research on COA’s. Currently, the research literature confirms what we knew 10 years ago: Some COA’s have problems, and some do not. Some COA’s with childhood problems grow into well-adjusted adults, whereas some of them do not. Likewise, some COA’s without childhood problems have a normal adulthood, and then again, some of them do not. Researchers are trying to sort out how risk is fulfilled or avoided in these groups and determine how risk changes over time. Achieving scientifically accurate predictability could help direct how we apply limited prevention dollars.

As already noted, some of the more recent COA studies have benefited enormously by introducing the methodological perspectives of the developmental psychology and developmental psychopathology fields, where the question of continuity and discontinuity of normal and problem behaviors has been examined. Unfortunately, not all COA studies have incorporated such developmental perspectives, and these studies continue to muddy the field. Because children of different ages and stages vary qualitatively and quantitatively, research design and methodology must be adjusted appropriately.

**Sher:** From my perspective, COA research is part of the much larger issue of the effect that parental variables (e.g., parental genotypes [genetic makeup], parenting abilities, abuse and neglect, family milieu, stressors, marital conflict, and divorce) have on child development. Increasingly, people recognize that alcoholism is not a single discrete entity that can be studied outside of a larger social context. In addition, people realize that environmental and genetic effects are not easy to disentangle. Moreover, there is greater recognition that alcoholism is a developmental disorder in several important senses: Alcoholism (1) appears to arise out of normative adolescent/young adult drinking,^3^ (2) can affect the normal course of adolescent and adult development, and (3) often remits in response to important developmental transitions in adulthood. The persistence of alcohol problems in the face of life roles that are incompatible with abusive drinking patterns probably represents a particularly severe form of alcoholism.

***AH&RW:**** What is the state of your own research?*

**Jacob:** My own studies of COA’s have incorporated several of the features I mentioned in response to the previous questions and currently focus on COA’s as young adults. Most important, we want to examine the course of alcoholism from adolescence through young adulthood, particularly to try to identify individual and relationship variables that characterize different developmental pathways.

**Chassin:** My own project has been attempting to live up to the ambitious agenda set out by Sher (1991), Windle and Searles (1990), and Zucker (1994) by following a large community sample of COA’s and non-COA’s from adolescence to young adulthood. This is an important age period, because we can now begin to examine the adolescent antecedents of substance abuse and dependence outcomes. As might be expected, the rates of alcohol and other drug abuse and dependence are high among the COA’s in our study, but approximately one-half of the sample is free of these problems, allowing us to test the mediators and moderators of COA risk. We also are beginning an investigation of the young offspring of the young adults in our study. By extending our research to another generation, we are coming full circle developmentally to examine some of the early temperamental underpinnings of risk. We hope our findings will complement those of longitudinal studies that have examined similar developmental periods, such as Ken Sher’s study of college students and Bob Zucker and Hi Fitzgerald’s study of preschool- and school-age children.

**Sher:** In my own research, I am currently studying the factors that influence the course of alcohol use disorders in early adulthood. I am particularly interested in how various courses of alcoholism (e.g., a developmentally limited course that remits before age 25; an early onset, persistent course; and a later onset course) are affected by background variables (e.g., family history of alcoholism, childhood trauma, and temperament) and important life transitions (e.g., finishing schooling, entry into the work force, marriage, and parenting). In addition, I am interested in examining how these variables and transitions work independently and in interaction with each other to determine the course of alcohol use disorders. These disorders may be time limited, episodic, or chronic, yet we know very little of what factors differentiate them.

***AH&RW:**** What have been the greatest achievements or breakthroughs so far?*

**Sher:** At this point I am reluctant to say that we have had any major breakthrough since the Danish adoption study in the early 1970’s. Certainly many potentially important findings have emerged, but their ultimate significance is still unknown. Perhaps the most important finding is the fact that both alcoholics and their children represent extremely diverse groups and any generalizabilities about them are likely to be of limited validity. However, I’d like to have the opportunity to answer this question again in 10 years; I sincerely think we will be in a much better position to assess the evidence then.

**Johnson:** I think a great achievement has been the refinement of methodological techniques and increasing understanding of the complexities involved in conducting developmental research. I don’t think there has been any single finding that could be considered a “breakthrough.” The problems associated with the inheritance of alcoholism, living with an alcoholic parent, changing social conditions, and all the possibilities for interaction make a breakthrough a long time in coming.

***AH&RW:**** What is the future direction of COA research?*

**Johnson:** There are three possible directions for COA research. The first is to continue as we have, completing cross-sectional studies that eventually will give us some limited information about small COA subgroups. These studies can be useful, especially if they are done well and are culturally and developmentally appropriate. The problem with this type of research, however, is that it takes a lot of time and imagination to try to piece this mosaic of studies together to create a recognizable picture of a developmental trajectory of high-risk children.

Second, COA research could analyze existing longitudinal data sets. There are secondary longitudinal data sets rich with possibilities for understanding COA’s, and performing secondary data analyses of them would be a perfectly respectable alternative to conducting new longitudinal research. Although the problems of identifying parental history in these data sets may not be trivial, this approach is worth a try, given that research dollars for longitudinal studies appear limited.

A third direction COA research might take is to conduct intensive studies of children at risk and extensively examine them during pubertal transitions and into adolescence using genetic, environmental, and behavioral research techniques. We know that rapid biological, physiological, and psychological changes occur simultaneously during this period. In addition, adolescents make choices that can affect them over their life course, which could interact with their biological heritage in such a way that alcoholism may or may not be expressed, depending on each adolescent’s developmental trajectory.

**Chassin:** Telling the future is a tough assignment! I think that future directions will continue the trends of recent work. That is, because we have barely begun to scratch the surface in terms of empirically testing multivariate models of COA risk and resilience, I think that future research will continue to broaden multidisciplinary perspectives, integrating designs that have been used in the past by diverse groups working in isolation (e.g., integrating genetic designs and biological assessment with studies of family interaction and broader psychosocial factors). This trend probably will be reinforced by changes at NIH [the National Institutes of Health] to review all research grants across the board, not simply within each Institute, which will emphasize the value of a multidisciplinary perspective. I also think that because we have a much greater understanding (both conceptually and in terms of data analysis) of what is required to empirically test mediational models, we will see more sophisticated work in this area. In terms of psychosocial research, I think the level of analysis will broaden somewhat to include more community-level variables and demographic diversity as well as more rigorous attempts to evaluate preventive interventions.

**Jacob:** For much of the past two decades, family studies of alcoholism have been conducted by two relatively nonoverlapping research groups. One group consists of psychosocial researchers interested in family environmental variables (e.g., parent-child relationships and family rituals) that interact with social environmental variables (e.g., peer relations) as well as individual variables (e.g., deficient behavioral control) in accounting for the link between a family history of alcoholism and offspring outcome. The other research group consists of behavioral geneticists interested in estimating genetic contributions to alcoholism risk and in differentiating the remaining environmental influences into influences shared among siblings in the same family and influences unique to each sibling (Jacob and Leonard 1994; McGue 1994).

A rich literature of theory and findings has developed from the first research tradition, which has offered increasingly sophisticated models of alcoholism etiology; defined a number of key mediator and moderator mechanisms that may account for or qualify the impact of family history risk on offspring outcome; and produced a number of high-quality, longitudinal data sets for testing alternative models of mediation and moderation. The major shortcoming of this research is one of indeterminacy or ambiguity of findings, however, because all efforts along this line have involved passive longitudinal designs (i.e., family studies) in which the separation of family genes from family environments is not possible. In contrast, behavioral genetic studies of the past 20 years have offered an increasingly persuasive argument that genetic influences ultimately account for 50 to 60 percent of the variance in alcoholism risk and that shared family environmental effects can only partly explain the remaining variance. At the same time, the strength of this conclusion and the extant behavioral genetic literature on alcoholism reflect several notable limitations. First, family and nonshared environmental influences have been poorly articulated and measured by behavioral geneticists. Second, we know very little about how environmental influences mediate and moderate genetic effects (i.e., the nature of gene-environment correlations and gene-environment interactions) in relation to alcoholism etiology and course. Third, researchers have not explored the impact of childhood and young adult events and behaviors (e.g., educational and occupational achievements, friendship networks, and marriage) in qualifying and/or explaining alcoholism risk within a genetically informative research design.

In light of these considerations, it seems imperative that future research on alcoholism etiology in general—and COA’s specifically—move toward an integration of behavioral-genetic and psychosocial perspectives to address unanswered questions. In particular, such efforts will need to learn more about gene-environment correlations and interactions that characterize the development and expression of alcoholism, drawing on highly informative behavioral-genetic research designs. Beyond simply estimating the strength of genetic-environmental influences, however, I hope that future research can identify and clarify genetically and environmentally based influences that account for the manner by which a family history of alcoholism predisposes people to alcoholism outcomes and that increase or decrease the likelihood of adverse outcomes among high-risk individuals. The key to such studies will involve (1) integrating the best thinking found in the psychosocial and behavioral-genetic literatures, (2) attempting to move beyond issues of “either/or” and toward questions regarding conditions by and under which complex behavioral patterns develop, and (3) relying on an overriding developmental framework within which course and outcomes can be better understood.

**Schuckit:** In the future, COA research will continue to place more emphasis on longitudinal studies measuring multiple domains simultaneously. We also will expand our measures of environmental influences. In addition, as we make progress in understanding specific genes that influence the risk for alcoholism, we hope to develop more precise prevention strategies by molding programs to meet the specific vulnerability involved.

Because alcoholism is such a complicated disorder, however, I think it is unlikely that we will reach the point where we know that a certain characteristic definitely will lead to alcoholism. To produce this syndrome, many different biological characteristics interact with the environment, and each one explains a relatively small proportion of the risk. We are becoming increasingly sophisticated in our understanding of the biological characteristics of alcoholism, and we are also increasing the sophistication of our understanding of environmental factors. At this point, we can identify who is at higher or lower risk, but we cannot say absolutely who will and who will not develop alcoholism. This realization is humbling, but it is also reassuring in that it is unlikely that specific findings can be used to discriminate against people (e.g., in regard to job or insurance opportunities). Nevertheless, in the final analysis, the more we know about each factor that enhances the risk of alcoholism, the greater our ability to develop more effective prevention methods will be.

## Footnotes

1 According to Brown (1988), “codependent” is a term that is often applied to people who organize their lives around other people and/or things; codependency becomes dysfunctional when an inability to regulate closeness and develop autonomy occurs. Codependency also has been noted as a pattern of immature adaptive mechanisms that frequently exists in alcoholics, their spouses, and their children (Cermak 1986).

2 *Editor’s note*: The term “affect regulation” refers to emotional stability (or lack thereof); the term “pharmacologic vulnerability” refers to an enhanced susceptiblity to the effects of a medication, drug, or, in this case, alcohol.

3 *Editor’s note*: That is, drinking behavior consistent with societal norms for adolescents and young adults.
